# Completing the “Nurses back to healthcare” training and returning to professional work: a qualitative study of nurses’ experiences

**DOI:** 10.1186/s12912-025-03281-9

**Published:** 2025-07-01

**Authors:** Kadri Kööp, Mare Tupits

**Affiliations:** Chair of Nursing, Tallinn Health Care College, Kännu 67, Tallinn, 13418 Estonia

**Keywords:** Nursing shortage, Returning nurse, Continuing education programme, Return to work

## Abstract

**Background:**

A substantial problem in the healthcare system is the lack of healthcare workers, especially nurses. An adequate number of nurses would reduce errors caused by work overload. The “Nurses back to healthcare” training is designed to alleviate the nursing shortage by providing an opportunity for nurses who are currently not working in the profession to re-enter healthcare. By reintegrating these experienced professionals, the programme helps to alleviate the nursing shortage, thereby improving patient outcomes and reducing strain on the existing workforce.

**Objectives:**

To describe nurses’ experiences of completing the “Nurses back to healthcare” training and returning to professional work.

**Design:**

A qualitative, empirical, and descriptive design.

**Participants:**

The participants were nurses (N = 17) who completed the training.

**Methods:**

Semi-structured interviews were conducted to collect data, and an inductive content analysis was performed to analyse data. Subject recruitment and data collection occurred concurrently until no new information emerged, indicating that saturation had been reached.

**Results:**

The nurses’ experiences of completing the training included experiences with starting studies, satisfaction with the training, training-related challenges, the type of support received, and recommendations for improving the training. The experiences of returning to professional work included experiences related to job search, work reintegration, support, and considerations for work reintegration. All 17 participants indicated a generally positive assessment of the training. However, 6 participants considered the volume of theoretical knowledge too extensive, and 10 participants suggested increasing the volume of practical activities.

**Conclusions:**

The “Nurses Back to Healthcare” training programme is essential in reintegrating nurses into the profession. Tailoring the programme to meet individual learning needs and adopting flexible approaches can significantly enhance its effectiveness. Participants with less professional experience and longer gaps since their studies require increased practice time, support in adapting to technological developments, and emotional support to address feelings of insecurity. Facilitating their transition back into the workforce also involves guiding them towards appropriate employment opportunities and offering flexible working conditions that consider their personal needs and prior experience.

**Supplementary Information:**

The online version contains supplementary material available at 10.1186/s12912-025-03281-9.

## Introduction

Nurses are vital to healthcare and form the largest section of the healthcare profession [1]. According to the World Health Statistics, there are approximately 29 million nurses and midwives globally, and by 2030, 32.3 million nurses and midwives will be needed [2]. The lack of healthcare workers, especially nurses, is a substantial problem in the healthcare system. Due to nursing shortage, nursing workload has increased [3]. Consequently, an increase in nurses’ workload increases the mortality rate of inpatients [4, 5]. An adequate number of nurses would reduce errors caused by work overload and increase patient satisfaction [1, 4].

High workloads, burnout, and job dissatisfaction influence nurses’ intention to leave the profession [6, 7]. Nurses’ intention to leave exposes patients to a greater risk of mortality [5] and impacts significantly healthcare organizations [6, 7]. If nurse turnover is not addressed, it will further increase the workload [5]. Nurses’ intention to leave can be tackled by organisational support and workload management interventions, such as implementing continuing education programmes, increasing staffing levels, and attracting new recruits [6].

One solution to increasing the number of nurses is to encourage currently unemployed nurses to return to the healthcare sector [8]. Returning nurses add value to the hospital unit and bring experiential knowledge to patient situations [9]. Inactive nurses returning to practice could use their skills and insights gained from previous similar practice situations, and recruiting returners is also a cost‑effective and efficient use of human resource management [10]. However, there are concerns about the competence of nurses returning to practice, as it is challenging for returners to achieve clinical competence [11]. Organisational support and return to practice programmes can efficiently help returners get prepared for professional nursing practice [12, 13].

Some countries have formalised return to work and practice programmes under government health departments. In refresher programmes, returners must develop their confidence and self-esteem to practise with a formalised learning structure [14]. Currently available comprehensive programmes have didactic, skill, and clinical components. These components help professionals update their skills and knowledge, gain confidence, and establish connections [9]. Return to practice programmes attract nurses who have professional awareness, clinical expertise, life experience, and transferable skills. These programmes may enable individuals to re-enter their profession at lower costs than conventional training programmes [15].

For training programmes to be effective and efficient, what aspects support individuals’ learning and ability to return to the workforce must be understood. Some reasons for not completing such programmes include difficulties completing the theoretical or placement component of programmes, unavailability of programmes in remote local facilities, inability to arrange clinical placement hours, family responsibilities, and health problems [14]. Returners have also been shown to have low self‑esteem and high anxiety and to be concerned about new working conditions and changes in organisational demands due to advances in technology and new responsibilities [12, 14]. Returners have unique needs and should not be treated as pre‑registered graduated nurses [9, 12]. Nurses need to feel appreciated and understood as competent professionals and individuals [15].

Given that returners often have low self-esteem and clinical competence [12], it is important to explore their experiences and needs regarding the refresher programmes, to utilise the time and resources of the training effectively and ensure that graduates are prepared to cope when they return to practice.

### “Nurses back to healthcare” programme in Estonia

In 2014, the “Nurses back to healthcare” project was planned in Estonia in cooperation with the Ministry of Social Affairs and Tallinn Health Care College. The rationale for launching the project was the fact that there were 6.6 nurses available per 1,000 inhabitants in Estonia, although the national goal was nine nurses per 1,000 inhabitants [16].

According to the “Health Care Services Organisation Act” in Estonia, professionals recognised as health care providers include doctors, dentists, nurses, and midwives, provided they are registered in the national register of health care professionals [17]. This register was established in 2002 by government regulation [18]. In Estonia, registered healthcare professionals are not required to undergo re-certification. However, individuals who obtained their qualification prior to the establishment of the national register, or who failed to register upon graduation, are often unregistered and thus ineligible to practice. To obtain registration in the national register of health care professionals, they must pass both theoretical and practical examinations, which can be particularly challenging for those who have been away from professional practice for extended period. In this context, the “Nurses back to healthcare” training programme supports them in preparing for and successfully passing these examinations [8].

The criterion for entering the programme was possession of a certificate confirming completion of a nursing curriculum; prior work experience was not required. In Estonia vocational higher education in nursing was introduced in 1996, replacing the earlier system of vocational training in medical schools. In the 2005/2006 academic year, medical schools were reorganised into applied higher education institutions, and nursing education was aligned accordingly. According to the Higher Education Act of 2019, graduates of applied higher education are awarded a bachelor’s degree [19]. Therefore, the educational level of participants in the “Nurses Back to Healthcare” project is primarily determined by the year they completed their nursing education.

The curriculum of the “Nurses back to healthcare” training comprises 30 ECTS credits, equivalent to 780 study hours, with one credit corresponding to 26 hours of student work. These credits are distributed as follows: 18 credits for general subjects, 6 credits for clinical practice, and 6 credits for Estonian language studies. General subjects include pharmacology, basics of nursing, nursing process and patient education, first aid, and specific subjects of the nursing profession - clinical nursing, mental health nursing, child nursing and primary care nursing. Students practice various nursing interventions and consolidate skills through situational tasks in a simulation environment. Following theoretical studies, students complete 156 hours of clinical practice in healthcare facilities [20]. They are allowed to choose their internship field based on personal interests and preferences, with options including various hospital departments, family health centres, and school healthcare settings. The course is structured in a cyclical format, conducted three days per week over five months. It concludes with both theoretical and practical examinations, the successful completion of which qualifies students for registration in the national register of healthcare professionals [8].

The “Nurses back to healthcare” project, initiated by the Estonian Ministry of Social Affairs, has proven successful, bringing over two hundred nurses back to healthcare in cooperation with Tallinn and Tartu Health Care Colleges [21]. A survey evaluating the project revealed that most nurses who completed the training were either already working or intended to work as nurses [8]. Thus, there is a target group that has completed nursing education and are interested in working as nurses, but who, for various reasons, are not currently employed in healthcare.

### Objective and research questions

Based on the abovementioned context, a qualitative study was conducted. This study sought to describe nurses’ experiences of completing the “Nurses back to healthcare” training and returning to professional work. The findings are intended to inform necessary changes and improvements to the training curriculum and support participants in starting work. Based on the aim of the research, the following research questions were formulated:

- How do nurses describe their experiences during the “Nurses back to healthcare” training programme?
- What factors influence the successful reintegration into professional work after completing the training programme

## Methods

### Design

This study adopted a qualitative and descriptive design. The essence of a qualitative methodology is that there are multiple perspectives about a phenomenon of interest, and these perspectives are inductively derived or discovered from people with personal experience regarding such a phenomenon [22]. Herein, a qualitative design was chosen to describe the present topic, as the study focused on the experiences of nurses. This approach helped in understanding the phenomenon more effectively. Semi-structured interviews were conducted for data collection, and an inductive content analysis was performed for data analysis [23]. Inductive content analysis is a method of analysing qualitative data that is well suited for use in health-related research, especially in relatively small-scale and non-complex studies [24]. The Standards for Reporting Qualitative Research checklist was used to report this study to enhance research transparency and quality [25].

### Sample and setting

In this study, purposive sampling was used, meaning that participants were deliberately selected to align with the research aim [26]. The inclusion criteria were completion of the nursing or midwifery curriculum and the “Nurses back to healthcare” training, return to professional work, and sufficient knowledge of the Estonian language, as the interviews were conducted in Estonian. Consent to participate in the interviews was obtained, and participation was voluntary.

The sample comprised 100 nurses who participated in the training since its launch in 2015. All participants had completed the programme at Tallinn Health Care College. The data were collected from December 2021 to October 2022. Potential subjects were contacted individually and consecutively. Consecutive sampling method was employed, whereby each subject meeting the eligibility criteria was selected over a specific time interval, or until a predetermined sample size was achieved [27].

Initially, potential participants were sent an invitation via email to participate in the research, followed by arrangements made via telephone to schedule the interviews, choosing between video platforms or face-to-face interactions. Participants had voluntarily provided their contact details (email addresses and phone numbers) at the onset of their training.

Interviews were successfully conducted with 17 active nurses. Some nurses did not meet the criteria because they had failed passing the theoretical and practical exams or had ceased working as nurses. Several nurses who completed the training were unreachable or declined to be interviewed, citing reasons such as a hectic lifestyle, not being in Estonia, or a lack of recall regarding the training details. Two participants initially agreed but subsequently became unresponsive to further communications. As data saturation depends on multiple factors [28], subject recruitment and data collection occurred simultaneously until saturation was reached. After conducting a total of 17 interviews, the researchers deemed the data collection sufficient, as the information gathered had reached saturation; no additional participants were approached thereafter.

### Data collection

Among those who participated in the project since its launch, a feedback survey was conducted following the completion of their training. This survey assessed their return to professional practice and gathered their insights on the curriculum content and the organisation of the studies. To obtain more comprehensive information and gain deeper insights, a semi-structured interview plan was developed based on the survey questions and literature review. According to Adeoye-Olatunde and Olenik [29], semi-structured interviews enable focused discussions while providing investigators the autonomy to explore pertinent ideas that may arise.

The semi-structured interview plan was developed for this study and consisted of introductory questions regarding participants, followed by the experiences linked to completing the “Nurses back to healthcare” training and returning to professional work (Appendix 1). The pilot interview was conducted with one participant who completed the training in the first year of project implementation. Piloting to test the interview questions was conducted in December 2021. As no modifications were necessary to the interview plan following this initial session, the data from the pilot interview were incorporated into the main study database.

The interviews were conducted from December 2021 to October 2022. Because of the COVID-19 pandemic, most interviews were performed through online platforms. The last three interviews were conducted face-to-face once pandemic-related restrictions were lifted. The majority of the online interviews (n = 12) were conducted during the subjects’ leisure time, typically at their homes. Two of the online interviews occurred during the subjects’ working hours and were conducted at their places of employment. Additionally, the three face-to-face interviews were held in public spaces that were convenient for the participants and took place during their free time. The interviews were conducted individually, and participants had no contact with each other.

A sample size is considered sufficient when further exploration does not reveal new characteristics within categories; this phenomenon is called data saturation [30]. Herein, the data began to become saturated after the 15th interview. Two more interviews were conducted to refine the initial categories. The average length of the interviews was 20 minutes; the shortest and longest durations were 13 and 27 minutes, respectively. All interviews were recorded and transcribed as soon as possible using Microsoft Word.

### Data analysis

In the current study, an inductive content analysis was used because there was limited information about the phenomenon. In qualitative research, data gathering and analysis frequently occur simultaneously [31]. Herein, data were analysed immediately after the first interview.

Initially, the researchers individually read the transcribed interviews multiple times and identified meaningful units relevant to the research phenomenon. Then, the researchers collectively discussed the meaningful units and formulated them into appropriate substantive codes. The categorisation process was conducted jointly by the researchers. Once open coding was completed, the researchers grouped codes with similar meanings which were related to the same phenomenon [26]. The grouped codes were entered into an abstraction table created in Google Sheets, and subcategories were formed. Then, the subcategories were further grouped into broader categories, and finally, main categories were established. The grouping process continued until the findings were coherent and logical [23].

### Ethical considerations

The study followed the ethical principles of the Declaration of Helsinki [32] and permission to conduct the study was obtained from Tallinn Health Care College (no. 1–16/358, issued on 21.06.2021) and the Research Ethics Committee of the National Institute for Health Development (decision no. 930, issued on 28.10.2021).

Interviews may cause emotional distress when participants are asked to reflect on past experiences, and they may have concerns about the confidentiality of their personal experiences [26]. To prevent this, a comprehensive informed consent form was prepared to ensure that participants clearly understood the purpose of the interview, the procedure, their rights, and how their data would be used. All participants signed the participant information and informed consent forms. These forms were sent beforehand for digital signing and participants could withdraw their participation at any time.

In qualitative research, the relationship between the researcher and the participant is closer than in quantitative research [26]. It is therefore important to maintain a trusting and respectful relationship while maintaining professional boundaries [33]. To support this, a neutral and supportive interview environment was created to encourage honest and open communication. Participants were also informed that they could contact the researchers at any time, should they wish to do so.

According to Elo et al. [34], authentic quotations may be used to increase the trustworthiness of research. In this study, quotations were used without specifying any person or place names to ensure anonymity. Anonymity was also ensured by coding the interviews by numbers (e.g. I1 for the interview conducted first, I2 for the interview conducted second). Special care was taken to maintain privacy and confidentiality when handling sensitive interview data. For this purpose, access to the collected data was restricted to the researchers and were securely stored using encrypted cloud storage.

## Results

### Sociodemographic data

A total of 17 nurses participated in the study. All participants were women. The youngest participant was 45 years old, while the oldest was 65 years old. The average age of the participants was 52 years. One held a master’s degree; three held a bachelor’s degree; 12 received applied higher education; and one received high school education with a vocational speciality. Table 1 illustrates the distribution of age and education level of the participants. All participants had returned to professional work after completing the training.

**Table 1:** Distribution of participants’ age and education level

Age	Number	Education level	Number
41–45	2	Master’s degree	1
46–50	7	Bachelor’s degree	3
51–55	5	Applied higher education	12
56–60	2	Vocational speciality	1
>60	1		

### Nurses’ experiences of completing the training

Two main categories emerged: nurses’ experiences of completing the “Nurses back to healthcare” training, and their experiences of returning to professional work. The first main category – nurses’ experiences of completing the training – was composed of substantive codes, which were combined into 14 subcategories and five categories. The experiences related to starting studies, satisfaction with training, training-related challenges, type of support experienced, recommendations for improving training (Table 2).

**Table 2:** Nurses’ experiences of completing the training

Categories	Subcategories
Experiences related to starting studies	Professional work experience before training Previous reasons for leaving a professional job Reasons to begin the course
Satisfaction with training Training-related challenges	Satisfaction with the organisation of the training Satisfaction with new knowledge Satisfaction with clinical practice
	Theoretical challenges Practical challenges Socioeconomic challenges
Type of support experienced	Emotional support Professional support Material support
Recommendations for improving training	Improving the organisation of the course Wishes regarding the improvement of practical training

### Experiences related to starting studies

The work experience of the participants before the training substantially differed. Nine participants had been away from healthcare for many years, and four had minimal professional experience. Participants’ previous professional backgrounds varied greatly. Four respondents worked as beauticians, and three respondents worked in the financial sector. The roles of caregiver, masseur, and managerial position were each mentioned twice. Additionally, the defence forces, an emergency centre, and a pharmaceutical company were each mentioned once as former workplaces. One respondent was already working as an assistant nurse. In the context of this category, school graduation and internship refer to the completion of basic nursing education, which was preceded by a pre-diploma internship at a healthcare institution.

*“… I just finished school, did an internship, and after that, I went to work in the aviation industry … then I was still very much past it…”. (I4)*

Raising children, changing residence, and experiencing burnout were mentioned as reasons for leaving professional work in the past. Seven nurses indicated that they suspended their nursing careers to prioritise family commitments, which they deemed more important than their professional roles in healthcare. Two participants mentioned that they experienced burnout when they started working at the hospital due to excessive responsibility and physical and mental stress. Over time, these individuals recognised that their departure from healthcare might have been preventable with adequate support and increased awareness of the early warning signs of burnout.

*“… I was 19 when I went to work. I worked for a year … then I realised that I was just burnt out…”. (I6)*

Nine participants indicated the need to register as healthcare workers as the reason for enrolling in the training. Only those healthcare workers registered in the national register are permitted to provide healthcare services. To be registered as a healthcare worker, it was necessary to pass a nurse’s theoretical and practical exam, which is difficult to successfully complete independently, especially after a prolonged absence from the profession.

Seven participants cited their vocation and love for nursing as the main reason for starting the training and considered their identity and self-definition as related to nursing. They described nursing as their true passion, their original calling, and felt they still had something to contribute to patients’ care. Other reasons included the desire for financial stability, career change, or life transitions.

*“… I remembered that the one place I have felt the safest has always been healthcare, and that was the motivation for returning…”. (I8)*

### Satisfaction with training

The participants indicated a general positive assessment of the training. For all of them, the training was a life-changing experience, as it provided them with an opportunity to re-enter healthcare. Ten participants were satisfied with the organisation of the training and the human-centred approach. One participant noted that the time was used to its fullest potential, while another praised the lecturers for their effective delivery of content. Three participants confirmed that they would not have been able to pass the registration exam and return to healthcare on their own.

*“… The course is very necessary … for example, I wouldn’t have been able to work as a nurse otherwise … everything was still very interesting for me…”. (I8)*

Participants reported gaining new and relevant knowledge, as nursing roles, interventions, and technologies had changed significantly over time. NANDA’s nursing diagnoses, nursing plans, documentation related to nursing activities, and information technology programmes were new for eleven participants. In the context of this research, references to “NANDA” by the subjects pertain specifically to the NANDA-I classification of nursing diagnoses. NANDA International is an organisation that facilitates the development, dissemination, and use of standardised nursing diagnostic terminology [35].

*“… Nursing diagnoses, because I graduated in 1997 and then there was no talk about them … I learnt from the training that nursing diagnoses and NANDA exist…”. (I7)*

Eleven participants shared several positive experiences in practice. Active participation and involvement provided an opportunity to practise extensively. Further, clinical practice was considered useful and versatile, as it could be conducted in different settings, thus providing diverse experiences. One participant admitted that they went to the internship with insufficient skills, but the one-month internship gave them confidence.

*“… Clinical practice was ideal in the sense that it was possible to do practice in several places, that is, it was not necessary to go to one institution for 156 hours…”. (I13)*

### Training-related challenges

Four participants experienced negative emotions when acquiring knowledge, as certain topics were too science-based and academic for them. As the electronic nursing documentation system is still under development in Estonia, not all participants had the opportunity to use NANDA-I classification system during their practice, which may be the reason why six participants considered its extensive coverage in the course disproportionate. Six participants also mentioned that the overall volume of theoretical knowledge was too large, so there was limited time for practising manual activities.

*“In my opinion, there was even too much of the theoretical side, and some things began to repeat themselves”. (I14)*

Four participants indicated that they were given too much responsibility and workload during clinical practice, and expressed concerns about their readiness for the responsibilities entailed. One participant found the practice in the emergency department to be a very traumatic experience, which might have been avoided in a calmer environment.

*“… Sometimes, I was completely alone … I had to do procedures … I had to answer the phone … I had to answer emails … I had to do what the doctor wanted to do for the patient … I did practically everything…”. (I4)*

According to the participants, it was difficult to balance training with family life. Three participants had young children, and found it challenging to manage everything logistically. It was also difficult to plan time alongside work and to make ends meet financially.

### Type of support experienced

Emotional support from others was highlighted as a significant component of their educational experience. The participants noted that coursemates, relatives, and family members were supportive. The availability of reliable help and support when needed was deemed essential. Moreover, the learners’ prior knowledge and personal motivation were recognised as key contributors to support their learning.

*“… A nice group we had there, that we held and supported each other. We were not like competitors, but companions, so that we found good words and morally supported each other”. (I12)*

Professional support from the college and internship base was also considered important. Participants identified accessible study materials, an optimal training duration, and employer support as crucial factors that facilitated their learning. Nine participants considered the teachers as helpful, professional, and supportive and the school’s attitude as generally positive. Conversely, three participants found their teachers unsupportive.

*“… There was a woman who you could turn to in case of questions … everything was nicely supported and there was help … everything was done very nicely by the school…”. (I5)*

During the internship, twelve participants received support from their colleagues and supervisors and considered clinical practice as a generally good and supportive experience. Conversely, four participants pointed out the lack of support and motivation from supervisors. One participant emphasised that the internship base must be patient and supportive, as they have been away from professional work for a long time.

*“But this nursing manager was absolutely not interested. When you went there, she said, ‘Hello, ahah, you’re here’, and when I left, it was exactly the same”. (I10)*

Material support was also considered important. The participants received scholarship during the training, which assisted them in managing their financial obligations but also served as a significant motivational factor for their participation in the training programme.

*“… There was a scholarship from the school, which was a great help. It was a great support…”. (I4)*

### Recommendations for improving training

Ten participants suggested to increase the volume of practical and manual activities and highlight their importance in the training. In the theoretical part, suggestions were made to pay more attention to primary healthcare, the role of family nurses in assessing patients’ health, and first aid instruction. There was a preference among three participants for more individualised practical training, as opposed to group work, due to a perceived insufficiency in practicing certain procedures. Six participants also suggested that the duration of the training could be extended and the approach more personalised. Three participants suggested increasing the use of simulation rooms, and one respondent thought that students or newly graduated nurses could be involved as instructors for practical procedures.

*“… We ran out of time … the training could have been longer … it could be, for example, half a year … there could have been more practical and individual learning … give more time to individual work … to give opportunity to use practice classrooms in free time … it seemed to be missing…”. (I3)*

Ten participants wanted to extend the internship period because it was difficult for them to combine internship with work. The practical component was fast-paced, and the opportunities to try things out on their own were limited due to time pressure. At the same time, four participants believed that learning occurred mainly on the job and considered a one-month internship to be sufficient.

### Nurses’ experiences of returning to professional work

The second main category – nurses’ experiences of returning to professional work – comprised substantive codes, which were combined into nine subcategories and three categories. The experiences related to job search, work reintegration experiences, support and considerations for work reintegration (Table 3).

**Table 3:** Nurses’ experiences of returning to professional work

Categories	Subcategories
Experiences related to job search	Supporting factors of finding a job Hindering factors of finding a job
Work reintegration experiences	Safe adaption when returning to work Satisfaction with current nursing job Challenges in returning to work
Support and considerations for work reintegration	Need for support in returning to work Experience of being qualified for healthcare work Aspects related to age Recommendations for improving work reintegration

### Experiences related to job search

Twelve participants had positive experiences with finding a job. Everything was resolved as expected, and no difficulties were experienced. Five participants received job offers to work in the same departments where they had completed their internships. Two participants shared that knowing different foreign languages was useful when finding a job. Given that these internships could span multiple locations, participants were presented with several employment options upon completion of their training. One participant mentioned having three job offers after completing the course.

*“… The nurse there really supported me and wanted me to come to work…”. (I4)*

Conversely, three participants found it difficult to find a job. They had no prior experience working as nurses, which made employers hesitant to hire them. Two participants were offered a position that required too much responsibility and was therefore refused.

*“… This responsibility seemed a bit big at that moment, and that’s why I didn’t dare to accept it…”. (I2)*

### Work reintegration experiences

The first work experience was made positive by the presence of a mentor when starting work, training based on the department, and amount of responsibility based on competence.

Thirteen participants had found an opportunity in healthcare that suited them and provided job satisfaction. They were satisfied with the working conditions and environment. Motivation from and teamwork with co-workers and a suitable work schedule were considered important.

*“… Let’s just say it’s 100% a dream job … I am very satisfied with the team, motivation, work schedule, and soon it will be 6 years…”. (I1)*

Various challenges were also experienced when returning to professional work. On one hand, they faced excessive responsibilities; on the other, they experienced a lack of patient-centeredness and autonomy in their roles. For instance, two nurses felt that they were perceived more as doctors’ assistants than as independent healthcare professionals. Additionally, they reported a deficiency in specific skills that were not covered during the training, leading to difficulties in using monitors and computer programs effectively.

*“… Patient-centredness is currently still a beautiful hope rather than a reality…”. (I6)*

### Support and considerations for work reintegration

Seven participants felt enthusiasm when starting work and did not need additional support. On the other hand, five participants experienced emotional difficulty due to insufficient experience and practical skills and the need to receive psychological support. One participant’s experience was that, since no one supported them, they didn’t dare to go to work.

*“The whole team was very supportive, and I had a lot of enthusiasm myself, so that’s great”. (I9)*

Seven participants mentioned that finding a job was supported by their own interest in looking for one and that they must be active and self-confident. Two participants expressed a desire for job placement assistance as part of the project, hoping to secure guaranteed employment in settings where they are truly needed and expected after completing their training.

*… In the old days there were referrals, maybe it’s time to restore such a system … I think it would help if it was already known that I would finish this program and that I would be expected somewhere … it would ensure that there is a point in studying in this course…”. (I13)*

The willingness to start working was also influenced by the fear of responsibility and lack of confidence. Five participants felt insecure in their manual actions, which they rated as weak. They shared that the lack of practical skills and experience with medicines caused difficulties when returning to work.

*“I wasn’t sure of myself. This manual activity, practical activity, I was still very, very weak”. (I12)*

The interviews revealed that age was an influencing factor of getting a job. Regarding age, there are both advantages and prejudices associated with it that influence perceptions and opportunities. Self-confidence and self-esteem could decrease with age, affecting decisions to change jobs. On the other hand, older participants pointed out that people at their age leave their jobs less often, while younger nurses are more likely to leave. One participant mentioned that if you are older, it is automatically assumed that you have extensive experience, but this may not be the case, and it creates pressure.

*“… But I’m 55 now. When I was 50, I came to work at a family health centre. I thought, well, at this age, is it worth doing it or not…”. (I1)*

To improve work reintegration, it was recommended to assign participants to a specific workplace, provide a probationary period when starting work, gradually increase the workload, and offer flexible working conditions. One participant suggested that returners to be allowed to work as assistant nurses during the training and recommended that more attention be paid to candidates’ backgrounds during admission interviews. They emphasised the importance of high internal motivation among those admitted to ensure the programme benefits those who genuinely intend to return to nursing.

### Facilitating and Hindering Factors

In summary, the main difficulties experienced during the training included balancing it with work and family life, and, at times a lack of support. Theoretical difficulties were perceived as stemming from an excessive emphasis on scientific theory, while practical challenges involved limited time for hands-on training and an overwhelming responsibility and workload in clinical practice. Consequently, these challenges led to uncertainty when applying for internships and jobs, and in some cases, to fear of accepting job offers.

Conversely, successful competence restoration was supported by the well-organised training programme and the valuable knowledge provided, along with opportunities to consolidate practical activities and undertake internships in various departments. A key factor in successful reintegration was the availability of diverse forms of support, starting with emotional support from lecturers, supervisors, peers, and family, and continued support from mentors and colleagues upon returning to work. Internal motivation and material support also played crucial roles. Return to work was further facilitated by department-specific training, a balanced level of responsibility, and flexible working conditions. The main factors that either facilitated or hindered the completion of training and return to work are presented in Table 4.

**Table 4:** Fascilitating and hindering factors in completing training and returning to work

Main Category	Facilitating Factors	Hindering Factors
Nurses’ experiences of completing the training	Useful new knowledge Good study organisation Hands-on experience Versatile practice in different settings Supportive teachers and supervisors Emotional support from groupmates and family Material support Learner’s internal motivation	Too scientific theoretical part Lack of applicability of NANDA nursing diagnoses Excessive theoretical content Limited time for practicing manual activities Too much responsibility during clinical practice Excessive workload during clinical practice Unsupportive teachers and supervisors Balancing with family and work responsibilities
Nurses’ experiences of returning to professional work	Internship base as a future workplace Foreign language skills Mentor’s support Department-based training Responsibility according to competence Satisfactory working conditions Teamwork with coworkers Internal motivation	Difficulty in finding job due to lack of experience Too much responsibility Lack of patient-centeredness Lack of autonomy Deficiency in manual skills Difficulties in using technology Lack of psychological support Lack of guaranteed employment Lack of confidence

## Discussion

The present study sought to describe nurses’ experiences of completing the “Nurses back to healthcare” training and returning to professional work. The analyses revealed diverse motivations and reasons for starting the training as well as both satisfaction with the training and training-related challenges Different supporting factors as well as suggestions for the development of curricula were identified. Regarding returning to professional work, experiences with finding a job and satisfaction with current nursing job were described. Experience of being qualified for healthcare work, the need for support and employment-related difficulties were also expressed. The findings could drive the necessary changes and improvements to training curricula, supporting project participants in starting work.

The shortage of nurses is one of the biggest challenges for healthcare systems worldwide [13]. This shortage can result in situations where important nursing tasks are partially or completely unperformed, a phenomenon known as ‘missed nursing care,’ which in turn can lead to the intention to leave the profession [6]. This intention can be mitigated by increasing staffing levels through the recruitment of new employees and continuing education training [6]. One solution is to re-integrate nurses not currently working as nurses into healthcare but based on the experience of these nurses, it is difficult to independently pass the registry examination, which is a prerequisite for working as a nurse. The theoretical and practical training conducted within the “Nurses back to healthcare” project could help nurses prepare for and pass the examination.

In this study, the work experience of the nurses before the training was either mostly minimal or completely absent. The nurses had been away from healthcare for a long time and left healthcare a few years after graduating from school. Similarly, Kent [9] found that as much as 25% of young licensed nurses in the United States left within the first 2 years of employment. The majority of participants in this training had completed their nursing education 20 to 30 years prior, making younger nurses with extensive work experience relatively rare exceptions within the group. In the current study, raising children, experiencing burnout, and having too much responsibility were cited as the reasons for leaving a former professional job. Burnout was also mentioned as a reason for leaving the profession in the studies by Mbanga et al. [36] and Kelly et al. [37]. Similar to the report by Barlow et al. [38], the participants in this study returned to professional work after a longer break when the family’s circumstances and their roles at home had changed. Kent [9] reported vocation and love for nursing as an important reason for starting training and returning to professional work. Love for nursing was also mentioned by several participants in this study.

According to the participants of this study, nurses’ role in healthcare as well as nursing interventions and technology used for work had changed substantially over time. Howell [39] also pointed out constant innovation and changes in healthcare, causing stress and pressure among nurses to provide quality patient care. In the present study, NANDA’s nursing diagnoses and nursing plans were completely new to most participants who had been away from healthcare for a long time in addition to the duty of documentation related to nursing activities and use of related information technology programmes.

The participants preferred a longer duration of training and a more personal approach. They deemed the theoretical component of the training as sufficient but considered the teaching of NANDA’s nursing diagnoses as too extensive. Some participants suggested more practical and individual learning. Emotional support was considered important supporting factor during the training, but material support was also regarded as essential. Barlow et al. [38] also confirmed that it was important for returners that they would be funded during the programme.

According to Nantsupawat et al. [40], the work environment is an important factor that affects nurses’ job satisfaction. This study showed that the internship base was the first actual contact with the work environment after being away from healthcare, which played a substantial role in future decisions. For most participants, the practice in the internship base was interesting and satisfactory. The participants emphasised that supervisor and peer support were a prerequisite for the success of practice, similar to the report by McMurtrie et al. [14]. There were also negative situations reported during practice when the participants were left completely alone. McMurtrie et al. [14] highlighted that it is important to specify the minimum number of hours completed under the supervision of a supervisor. This means that supervised clinical practice must be a mandatory part of continuing education.

Most participants in the present study felt a lack of practical readiness for returning to work before the training program, but some of them remained uncertain even after completing the training. They rated their experiences as rather weak and limited. The participants would have liked to have gained more experience in manual activities and to have practised more during the training, which would have increased their readiness for returning to work. These findings are consistent with the report by White [41] that attention should also be paid to the emotional preparation of nurses in relation to professional work. According to some participants, the readiness for returning to work would be improved if they were referred to placements where employment is guaranteed upon completion of the project.

Upon returning to work, participants discovered that the computer programs were highly specialised, and participants identified a deficiency in specific skills not addressed during the training, which subsequently led to difficulties in the effective utilisation of monitors and computer programs. The participants would have liked training on this topic before returning to the labour market. According to Mohammadi et al. [12], nurses returning to work have low self-esteem and high anxiety due to the innovations and changes brought about by technological development. Therefore, training participants could be introduced to the most important programmes and equipment used in healthcare.

Several researchers [9, 10, 12, 15, 38] have emphasised that returning nurses could bring experience and maturity to nursing practice. Since the participants in this study were generally of mature age, but with limited experience, the support mechanisms must be tailored to meet their specific needs. Nurses of a more mature age are likely to remain in the profession until retirement, thereby contributing to a more stable workforce [42]. However, they might also experience a decline in self-confidence and self-esteem as they age, a concern corroborated by findings from this study where participants reported feeling insecure about their practical skills. The lack of practical training significantly impacts nurses’ preparedness for their professional roles. Without practical experience, nurses may struggle to apply theoretical knowledge effectively, resulting in decreased confidence and competence in clinical settings. This can result in higher rates of ‘missed nursing care,’ where essential tasks are not performed, ultimately impacting patient outcomes and increasing job dissatisfaction among nurses, as stated also by Azzelino et al. [6]. Consequently, there is a recognised need among participants to enhance practical training components rather than focus extensively on theoretical knowledge. However, the “Nurses back to healthcare” training providers emphasise the equally important theoretical understanding, and increasing the proportion of practical learning would necessitate extending the duration of the training programme, which in turn could require additional financial resources. In general, participants in the ‘Nurses back to healthcare’ project can be divided into two groups. The majority were older, with a longer period since completing their studies, often having less professional work experience and lower self-confidence, requiring longer internships and more individual support. The smaller proportion were younger, who had more recent training and, in many cases, more professional experience. They displayed greater self-confidence and considered the duration of the internship sufficient, without the need for additional support. Such a difference within the target group highlights the need for an individual approach. Rather than modifying the entire curriculum for all participants, training should incorporate an assessment of individual learning needs, with additional consultations and support offered as necessary. This represents an important new finding of the study that deserves to be highlighted.

To support the reintegration of inactive nurses, several countries have established comprehensive return-to-practice programmes. For example, Australia has implemented a return-to-practice programme that meets the requirements of the Re-Entry Registered Nurses Accreditation Standards, which outline the core competencies for assessing the performance of registered nurses [43]. Similarly, the Nursing and Midwifery Council in the UK has set standards for return-to-practice programmes that outline legal and entry requirements, supervision and assessment requirements, programme content and information on the credits [44]. Barlow et al. [38] had evaluated the return-to-practice programme in UK and recommended that returning nurses should be supported to achieve their objectives as smoothly as possible, which means responding to the individual learning needs and allowing flexibility for those who need longer period for independent study. Japanese researchers, Yamamoto et al. [10] add that forming a mutual support network among returning nurses can facilitate the exchange of experiences and provide mutual encouragement. They also argue for the creation of a family-friendly environment is crucial. The authors of current study agree with the multidimensional approach proposed by Yamamoto et al. [10] as essential for harnessing and retaining the valuable potential of these human resources.

The present survey revealed a general positive assessment of the training. For most participants, the training was a life-changing experience, as it was the only way to re-enter the healthcare profession. Scammell [45] noted that given the escalating nursing shortage, it is imperative to reengage as many registered nurses as possible, providing them with the necessary support to adapt to contemporary practice. Therefore, Yu et al. [11] recommended that re-entry training programmes be implemented to provide inactive nurses with the knowledge and skills required to improve their nursing competence.

### Strengths and limitations

The strength of this study is that it was based on a nationally initiated training programme and long-term experience with the implementation of the programme. The long-term background of the programme made it possible to ensure a sufficient number of participants who had enough relevant information for the study. Conversely, the limitation of the study is the constrained number of face-to-face interviews that could be conducted due to the restrictions imposed by the COVID-19 pandemic. Most interviews had to be conducted via a video platform, which did not allow a comprehensive observation of the emotions and body language of the participants. Restrictions due to COVID-19 pandemic may have affected the depth and duration of interviews, because face-to-face interactions typically foster a more trusting relationship, allowing subjects to be more forthcoming. Another limitation is the language barrier. One of the prerequisites for completing the training and participating in the interview was sufficient knowledge of the Estonian language. Not all participants sufficiently understood the content of the questions, and the interviewer had to explain the questions in a simpler way.

### Implications for practice and research

Improving reintegration programmes can benefit from the example set by the United Kingdom, where the number of hours spent in practical training placements varies depending on the time the participant has been away from the register, and the duration of the programme can range from three months to one year [46]. Therefore, the ideal training model is should not be fixed in terms of hours and duration but rather flexible and tailored to the individual learning needs of the returnee. To better balance theoretical learning, more topics related to primary healthcare and the work of family nurses could be incorporated, considering the feedback from participants, as returnees often envision themselves working in primary care. To enhance practical learning, the use of simulation rooms could be expanded, and additional consultations and individual hands-on practice opportunities should be offered. In the selection of teaching methods, individual learning should be preferred over group work. Smooth transition back to work can also be supported by direct referrals to job positions.

Long-term follow-up strategies, such as feedback surveys and focus group interviews among both returnees and employers are suitable. In future research, the experiences of nurses who have participated in similar training programmes in Estonia over the years could be studied using a quantitative method to obtain a broader overview at the national level, achieving generalisable findings.

## Conclusions

Nurses who have completed the “Nurses back to healthcare” training find it necessary and useful to return to the profession they love. Given the diverse backgrounds of the participants, the development of the programme should be tailored to their individual learning needs. Adopting flexible and personalised approaches can significantly enhance the effectiveness of reintegration programmes.

Participants with limited professional experience and a longer gap since their studies require additional practice time and more opportunities to develop their practical skills. They also need support in adapting to technological advancements, as well as emotional support to help them overcome feelings of insecurity. Facilitating a smooth transition back into the workforce involves guiding these individuals towards appropriate job roles and offering flexible working conditions that take into account their personal circumstances and prior professional experience.

## Electronic Supplementary Material

Below is the link to the electronic supplementary material.

Appendix 1 - Interview Plan

## Data Availability

The data that support the findings of this study are available from the corresponding author upon reasonable request.
