# Supplementary material for: Completing the “Nurses back to healthcare” training and returning to professional work: a qualitative study of nurses’ experiences
Source: BMC Nurs. 2025 Jul 1;24:781. doi: 10.1186/s12912-025-03281-9 (PMC12211176; doi:10.1186/s12912-025-03281-9)
Supplement: Supplementary file 1 — Appendix 1 - Interview Plan [file 12912_2025_3281_MOESM1_ESM.pdf]

INTERVIEW PLAN

I. Introductory Questions

Age

Education level

Employment status

In which year did you participate in the project "Nurses Back to Healthcare"?

II. Experiences with the "Nurses Back to Healthcare" Training

What were the main factors that influenced you to return to healthcare and participate in the training?

Which knowledge areas were most updated during the training?

What knowledge and skills should have been covered more during the training?

How do you evaluate the balance between theoretical topics and practical exercises – what would you need more of, and what less?

Who/what supported you during the training?

What difficulties did you experience during the training?

What additional support would you have needed during the training?

What are your suggestions regarding the curriculum structure and the organization of the studies?

What are your suggestions regarding the preparation and organization of the exam?

III. Experiences with Returning to Professional Work

What position are you currently working in, having been registered as a healthcare worker?

How long have you been working in your professional field since completing the project?

Who/what supported you in returning to professional work?

What difficulties did you face when returning to professional work?

What support would you have needed when returning to professional work?

How did the clinical practice fulfill its purpose – did it support your employment, or should something be changed regarding the practice?
